# Understanding children’s preference for park features that encourage physical activity: an adaptive choice based conjoint analysis

**DOI:** 10.1186/s12966-021-01203-x

**Published:** 2021-10-09

**Authors:** Jenny Veitch, Kylie Ball, Elise Rivera, Venurs Loh, Benedicte Deforche, Anna Timperio

**Affiliations:** 1 Institute for Physical Activity and Nutrition (IPAN), School of Exercise and Nutrition Sciences, Deakin University, Geelong, Australia; 2 Burwood, Australia; 3 Department of Public Health and Primary Care, Faculty of Medicine and Health Sciences, Ghent University, C. Heymanslaan 10, 9000 Ghent, Belgium; 4 Movement and Nutrition for Health and Performance Research Group, Department of Movement and Sport Sciences, Faculty of Physical Education and Physical Therapy, Vrije Universiteit Brussel, Pleinlaan 2, 1050 Brussels, Belgium

**Keywords:** Parks, Green spaces, Active, Children, Urban, Attributes, Preferences

## Abstract

**Background:**

Parks are a key setting for physical activity for children. However, little is known about which park features children prefer and which features are most likely to encourage them to be active in parks. This study examined the relative importance of park features among children for influencing their choice of park for engaging in park-based physical activity.

**Methods:**

Children (*n* = 252; 8-12 years, 42% male) attending three primary schools in Melbourne, Australia completed a survey at school. They were required to complete a series of Adaptive Choice-Based Conjoint analysis tasks, with responses used to identify the part-worth utilities and relative importance scores of selected park features using Hierarchical Bayes analyses within Sawtooth Software.

**Results:**

For the overall sample and both boys and girls, the most important driver of choice for a park that would encourage them to be active was presence of a flying fox (overall conjoint analysis relative importance score: 15.8%; 95%CI = 14.5, 17.1), followed by a playground (13.5%; 95%CI = 11.9, 15.2). For the overall sample, trees for climbing had the third highest importance score (10.2%; 95%CI = 8.9, 11.6); however, swings had 3rd highest importance for girls (11.1, 95%CI = 9.3, 12.9) and an obstacle course/parkour area had the 3rd highest importance score for boys (10.7, 95%CI = 9.0, 12.4). For features with two levels, part-worth utility scores showed that the presence of a feature was always preferred over the absence of a feature. For features with multiple levels, long flying foxes, large adventure playgrounds, lots of trees for climbing, large round swings, large climbing equipment, and large grassy open space were the preferred levels.

**Conclusion:**

To ensure parks appeal as a setting that encourages children to engage in physical activity, park planners and local authorities and organisations involved in park design should prioritise the inclusion of a long flying fox, large adventure playgrounds, lots of trees for climbing, large round swings and obstacle courses/parkour areas.

**Supplementary Information:**

The online version contains supplementary material available at 10.1186/s12966-021-01203-x.

## Introduction

Global evidence affirms the importance of regular physical activity for children for better physical health, including cardiorespiratory fitness, bone development and improved weight status, as well as enhanced mental, social, and cognitive health [1–3]. Despite these benefits, the majority of children worldwide do not achieve the recommended daily 60 min of moderate-to vigorous-intensity physical activity (MVPA) [4]. Evidence has shown that children’s low physical activity may track into adolescence and adulthood [5, 6], making physical activity promotion during childhood paramount for present and future health.

Research has shown that time spent outdoors is positively associated with children’s physical activity [7] and public parks have become well recognised as a key setting for facilitating physical activity in a natural outdoor environment [8]. Parks not only provide opportunities for social interaction, relaxation and contact with nature, but also offer a supportive environment through the provision of activity-conducive amenities, such as playgrounds for climbing and swinging [9], and grassy open space and sports features for active recreation [10]. In addition, parks are sometimes described as an antidote to counterbalance the technological saturation among children [11]. Further, research has demonstrated that exercise performed in nature (green exercise) may confer greater health benefits, such as reduced stress and improved emotional well-being and overall mental health [7], than activity in other urban settings [12].

Although the majority of children in Australia have access to a park near home [13], many parks are not often visited or well utilised by children [14, 15]. The nature of children’s physical activity whilst visiting parks has been shown to range from sitting/standing to more vigorous activities [9, 16–19], with higher intensity of physical activity observed when using sports courts, fields and playgrounds in contrast to lower intensity physical activity in shelters/picnic areas and water play areas [18, 20, 21]. Additionally, gender differences have been observed regarding the types of activities performed in parks [22], with girls being more likely to walk/run, be sedentary and use playgrounds, and boys being more likely to play active games and use sports facilities [18, 23]. It may be that the varied park use observed among children and between boys and girls reflects different preferences for park features that encourage park-based physical activity.

It has been suggested that appealing park features may be more important for encouraging park visits than park proximity or accessibility among children. For example, a recent study in Melbourne found that parents are willing to visit parks located further from home if they are equipped with relevant features, such as sports courts and ovals [24]. Quantitative and qualitative research has shown that playgrounds, shade, greenery, active recreation facilities, sports features (e.g., courts, fields), natural and water features, trees for climbing, open space, and sports programs are important for facilitating youth physical activity [13, 25–30]. However, the relative importance of different park features for promoting children’s physical activity remains unknown.

Choice-based conjoint analyses examine how people value different characteristics (e.g., park features) of a product (e.g., park) and identify which characteristics have the greatest influence on preference or choice. Among adolescents, the limited evidence available using conjoint analysis indicates that park maintenance and play and fitness equipment were most important for encouraging adolescents’ park visitation and park-based physical activity [31, 32]. However, the park features that are important drivers of children’s preferences may differ from those of adolescents [22], and to date no study has examined which park features are most important relative to other features, for influencing choice of parks for encouraging active park use among children. A potential long-term strategy for increasing physical activity is to create and refurbish parks with features most preferred by a range of different people, including children, to maximise active use of parks by everyone. This is especially critical when resources are scarce. This study used Adaptive Choice-Based Conjoint (ACBC) analysis to examine the relative importance of park features with respect to children’s preference for a park that would encourage them to engage in physical activity and explored differences according to gender. ACBC analysis is a quantitative market research method that examines how much people value specified features and different aspects of the features (feature levels) when making choice-based decisions between two options with a combination of features and feature levels [33]. In this study, this technique was applied to selected park features (i.e., swings) and park feature levels (i.e., large round swings, group swings in a circle, traditional swings, no swings).

## Methods

This research was part of a larger study (ProjectPARK) that examined park characteristics influencing park visitation, park-based physical activity and social interaction among children, adolescents, and older adults [34–38]. For this component of the study, children in grades 3-6 (8-12 years) attending primary schools in Melbourne, Australia completed a survey on iPads during school time between November and December 2019. The survey took 15-30 min to complete. ACBC analysis, using Sawtooth SSI Web Lighthouse Studio 9.8.0 (www.sawtoothsoftware.com.au), was used to identify the relative importance of selected park features for influencing choice of park for active park use. Approval to conduct the study was obtained from the Deakin University Human Ethics Advisory Group (94_2017) and the Department of Education and Training.

### Recruitment

Four primary schools that participated in an earlier phase of this project [38] were re-contacted to confirm their participation in this study. Three of these four schools agreed to participate in this study, one from each socio-economic status (SES) area (low, middle, and high) based on the Australian Bureau of Statistics Socio Economic Index for Areas (SEIFA) for postcode [39]. The principal or delegate from each school was asked to purposefully select grade 3-6 classes to avoid inclusion of children who had participated in the previous phase of the study [38]. Participating schools provided between four and seven classes. Plain language statements and parental consent forms were sent home with the students in selected classes (*n* = 488 students). Completed consent forms were returned by 281 students (63%); 13 students were absent on the day of data collection, five students had incomplete ACBC data, and 11 students were excluded due to participation in the previous study, resulting in a final sample of 252. Participation rates for eligible students from low, middle, and high SES schools were 25% (*n* = 62), 47% (*n* = 118) and 29% (*n* = 72), respectively.

Participants completed survey items, including age, sex, school year, dog ownership, number of days they were physically active for at least 60 min in in a typical week [40, 41], and time taken to walk from home to the park they visited most often. They also responded to items relating to their usual park visitation over the past 3 months: frequency and duration of park visitation; activities engaged in and activity levels when visiting parks; accompaniment at the park; frequency of meeting or talking to people they already knew, and to people they did not know, when at the park; and mode of transport used to travel to the park (see Table 1 for response options). These items have been used in previous studies with children of this age [38].

**Table 1:** Demographic characteristics of participants (*n* = 252)

Age, mean years [SD]	10.2 [1.4]
Sex, Male, n(%)	105 (41.7)
Dog owner, n(%)	85 (33.7)
School year level n(%)	
Year 3	85 (34.1)
Year 4	38 (15.3)
Year 5	28 (11.2)
Year 6	98 (39.4)
Usual frequency of park visit, n(%)	
≥ once per week	139 (55.4)
2 -3 times per month	53 (21.1)
≤ once per month	41 (16.3)
Haven’t visited a park in the past 3 months	18 (7.2)
Usual duration of park visit, n(%)	
< 30 mins	19 (7.6)
30 min to 1 h	96 (38.4)
> 1 to < 2 h	62 (24.8)
2 or more hours	55 (22.0)
Haven’t visited a park in the past 3 months	18 (7.2)
Usual activity levels during park visit, n(%)	
Mostly sitting	8 (3.2)
Mostly light activities	22 (8.9)
Mostly moderate activities	127 (51.2)
Mostly vigorous activities	73 (29.4)
Haven’t visited a park in the past 3 months	18 (7.3)
Usual accompaniment to the park, n(%)^a^	
No one (without dog)	45 (17.9)
Parent	166 (65.9)
Other adult	64 (25.4)
Sibling(s)	146 (57.9)
Friend(s)	146 (57.9)
Organised Group	47 (18.7)
Dog(s)	58 (23.0)
Haven’t visited a park in the past 3 months	18 (7.1)
Usual activities performed, n(%)^a^	
Walk	80 (31.7)
Walk the dog	63 (25.0)
Jog	83 (32.9)
Ride bike/scooter/skateboard	129 (51.2)
Ball games	123 (48.8)
Play on playground	161 (63.9)
Picnic/BBQ	76 (30.2)
Watch sport	35 (13.9)
Major event	52 (20.6)
Hung out with family	127 (50.4)
Hung out with friends	154 (61.1)
Relaxed	95 (37.7)
Café	37 (14.7)
Geocaching/outdoor treasure hunt	12 (4.8)
Virtual reality games	17 (6.7)
Haven’t visited a park in the past 3 months	18 (7.1)
Usual mode of transport to park visited most often, n(%)^a^	
Walk	181 (71.8)
Jog	62 (24.6)
Cycle	105 (41.7)
Public Transport	8 (3.2)
Car	127 (50.4)
Haven’t visited a park in the past 3 months	18 (7.1)
Frequency of talking to people in park never met previously, n(%)	
Never/rarely	137 (54.4)
Sometimes	59 (23.4)
Most of the time/always	38 (15.1)
Haven’t visited a park in the past 3 months	18 (7.1)
Frequency of talking to people in park that they already knew, n(%)	
Never/rarely	120 (47.6)
Sometimes	77 (30.6)
Most of the time/always	37 (14.7)
Haven’t visited a park in the past 3 months	18 (7.1)
Number of days of ≥60 mins physical activity per day in usual week, n(%)	
< 7 days/week	186 (74.7)
7 days/week	63 (25.3)

^a^Multiple responses allowed

In a previous phase of the ProjectPARK study [38], children (*n* = 274) rated images of 42 park features according to how likely it is that the features would encourage them to engage in park-based physical activity. The top ten rated features from that study (based on mean scores) were identified separately for both boys and girls equating to 11 features, as the features in the top ten varied by sex. Previous research among adolescents in Belgium showed that preference for park features can vary according to frequency of park visitation and engagement in physical activity [42], thus, we also calculated mean scores and rankings for each feature according to frequency of park visitation (i.e. visit ≥ once/week, visit < once/week) and whether or not participants met physical activity recommendations (i.e., ≥ 60 mins MVPA/day, < 60 mins MVPA/day) via self-reported days per week they performed at least 60 min MVPA/day. Two additional features were in the top ten when ratings were examined by these two sub-groups; large grassy open space was ranked 10th for those who reported visiting parks <once/week, and sports walls were ranked 8th for those who did not meet physical activity recommendations (data not shown). This resulted in the inclusion of written descriptions for a total of 13 features (see Table 2). Printed handouts showing images of examples of these features were also available to students whilst they were completing the survey. Each feature was presented with two levels - present or absent (e.g., sports goals, no sports goals); or three or four levels - high-to-low sequence order (e.g., giant slides, medium slides, no slides).

**Table 2:** Park features and feature levels

**Feature**	**Feature level**
1. Playground	i. Large adventure playground
	ii. Small playground
	iii. No playground
2. Obstacle course/parkour	i. Obstacle/parkour course
	ii. No obstacle/parkour course
3. Slides	i. Giant slides
	ii. Medium slides
	iii. No slide
4. Flying fox	i. Long flying fox
	ii. Short flying fox
	iii. No flying fox
5. Climbing equipment	i. Large climbing equipment
	ii. Small to medium climbing equipment
	iii. No climbing equipment
6. Waterplay	i. Waterplay area
	ii. No waterplay area
7. Trees for climbing	i. Lots of trees for climbing
	ii. Some trees for climbing
	iii. No trees for climbing
8. Swings	i. Large round swings
	ii. Group swings in a circle
	iii. Traditional swings
	iv. No swing
9. Outdoor fitness equipment	i. Outdoor fitness equipment
	ii. No outdoor fitness equipment
10. Grassy open space	i. Large grassy open space
	ii. Medium grassy open space
	iii. Little to no grassy open space
11. Interactive areas	i. Interactive areas
	ii. No interactive areas
12. Sports goals	i. Sports goals
	ii. No sports goals
13. Sports wall	i. Sports wall
	ii. No sports wall

A series of ACBC tasks were completed by all participants to identify preferences for features and feature levels for encouraging park-based physical activity. An ACBC survey is interactive as tasks are customised in real time based on individual preferences reported as participants progress through the survey [43]. In the previous rating study features were examined independently or in isolation, whereas with ACBC the features are examined conjointly. ACBC surveys tend to be realistic of people’s real life decision-making as they employ non-compensatory procedures [44]. Participants were required to complete each task before progressing to the next task. Firstly, respondents selected six features from the list of 13 that would be most likely to encourage them to be active in the park. If any of the six selected features had three or four levels, participants were required to select their most preferred level. For example, for the feature ‘slides’, participants were asked to select their preferred level: ‘giant slides’, ‘medium slides’, or ‘no slides’.

Respondents were then shown a series of six ‘screening’ questions where four parks were described; each park had a different combination of feature levels for the six selected features. For each park, participants were required to indicate if the park described ‘would’ or ‘would not’ encourage them to be active. Based on responses to these screening questions, the program identified if any particular feature levels were included in parks that were selected as ‘not encouraging’ them to be active. In those cases, participants were asked additional question(s) to indicate whether any of the identified feature levels would be ‘totally unacceptable’ and to select the one feature level that was most unacceptable. Similarly, when the program’s algorithm detected that certain feature levels were consistently included, respondents were asked to select the feature level that a park ‘must have’ to encourage them to be active when at the park. These ‘must have’ and ‘unacceptable’ questions determined whether specific feature levels were non-compensatory for choice and ensured that remaining tasks included features levels that best met each individual’s needs.

Participants were then shown a series of 13 ‘choice tasks’ that included descriptions of two parks. They were asked to select the park that was most appealing for encouraging them to be active. Each park had a different combination of the levels of the six features that had been identified in the previous sections as ‘possibilities’ (i.e., feature levels had not been marked as ‘unacceptable’). The “winning” park profile for each choice task tournament was included in the following rounds of the choice task tournament until the most preferred profile was determined [44]. Examples of each of the main tasks are included in Additional file 1. Pilot testing was performed with two girls and two boys aged 7-12 years, and minor amendments for clarity were made accordingly.

### Data analyses

Descriptive characteristics of the sample were calculated using Stata version 15 (Stata Corp., College Station, TX, USA). Data from the ACBC survey were analysed using Sawtooth SSI Web Lighthouse Studio 9.8.0. ACBC analysis yield two types of parameters: part-worth utilities and average relative importance scores [43]. A part-worth utility represents the preference for a level within each feature, with a higher, positive value indicating a greater preference for that level*.* For example, if the feature levels ‘giant slides’, ‘medium slides’ and ‘no slides’ had part-worth utility values of 20, 15 and − 5 respectively, this means that ‘giant slides’ were the most preferred level and ‘no slides’ were the least preferred level. To assist with interpretation, part-worth utility scores were zero-centred by the software. A low, negative value for ‘no slides’ does not indicate that this level was disliked but rather that it was the least preferred of all the feature level options. Part-worth utilities can only be compared within a given feature (i.e., cannot be compared to the preferred levels of other features).

Relative importance scores are presented as a percentage and represent which features have the greatest or least effect on choice, with greater importance scores reflecting greater effects on choice. Importance scores for a feature are the difference between the least and most favorable feature levels (i.e., range from the part-worth utility values for the levels for that feature). The importance scores are also ratio-scaled; for example, a feature with an importance score of 20% is twice as important as a feature with an importance score of 10%.

Individual part-worth utility and importance scores were estimated for the overall sample and according to gender with Hierarchical Bayes (HB) analyses [45]. For each part-worth utility and importance score, standard deviations, standard errors and 95% confidence intervals were calculated and presented graphically. Significant differences between levels of each feature (part-worth utilities) and different features (importance scores) were indicated by non-overlapping confidence intervals. The overall fit of a HB model was interpreted with Root LikeliHood (RLH) values ranging from zero to one, with a higher value indicating a better fit of the model. There were 40,000 iterations for the HB models, the recommended number for reaching successful convergence [43].

## Results

The demographic characteristics of the sample are presented in Table 1. Children (42% male) were spread across grades 3-6. More than 50% of participants reported visiting a park at least once per week, approximately 80% usually engaged in MVPA during their park visits, 66% were usually accompanied by a parent and 72% reported usually walking to the park they visited most often. More than half (54%) of children never or rarely spoke to people they had never met previously and 48% never or rarely spoke with people they already knew (48%) when in the park.

### Part-worth utilities

**Fig. 1:**
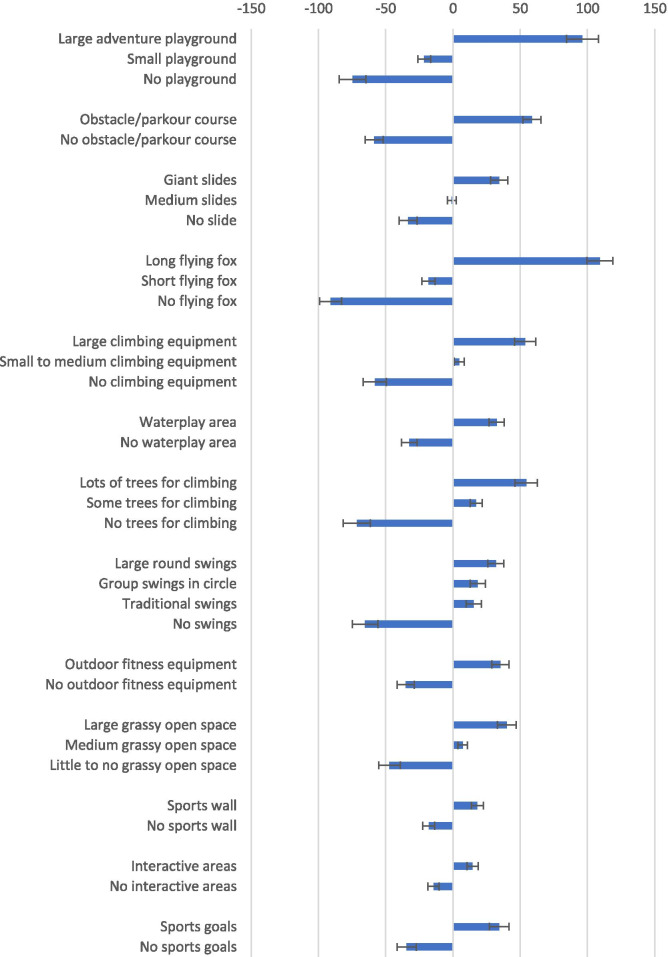
Overall average utilities for park features encouraging physical activity

The overall average utilities of the park features are presented in Fig. 1. For features with two levels, the presence of a feature was always preferred over the absence of a feature. For example, there was a higher preference for having a water play area (32.5, 95%CI = 26.8, 38.2) than having no water play area (− 32.5, 95%CI = − 38.2, − 26.8).

For features with three levels, the higher sequence order was always preferred. For example, having no climbing equipment had a significantly lower preference (− 58.2, 95%CI = -66.7, − 49.7) than medium climbing equipment (4.5, 95%CI = 0.8, 8.2), which again had a lower preference than large climbing equipment (53.6, 95%CI = 45.8, 61.5). These findings were consistent for boys and girls. For swings, large round swings had the highest part-worth utility score (31.8, 95%CI = 25.8, 37.8) overall, followed by group swings in a circle (18.3, 95%CI = 12.7, 23.9) and traditional swings (15.4, 95%CI = 9.8, 21.0) which were not significantly different from each other, and then no swings (− 65.4, 95%CI = -74.9, − 56.0). These findings were consistent for girls. However, for boys’ preferences for large round swings (23.94, 95%CI = 13.7, 34.2) and traditional swings (22.54, 95%CI = 12.1, 33.0) were similar, with lower preference for group swings in a circle (3.53, 95%CI = -2.1, 9.1) and no swings (− 50.0, 95%CI = -62.9, − 37.2).

### Relative importance scores

**Fig. 2:**
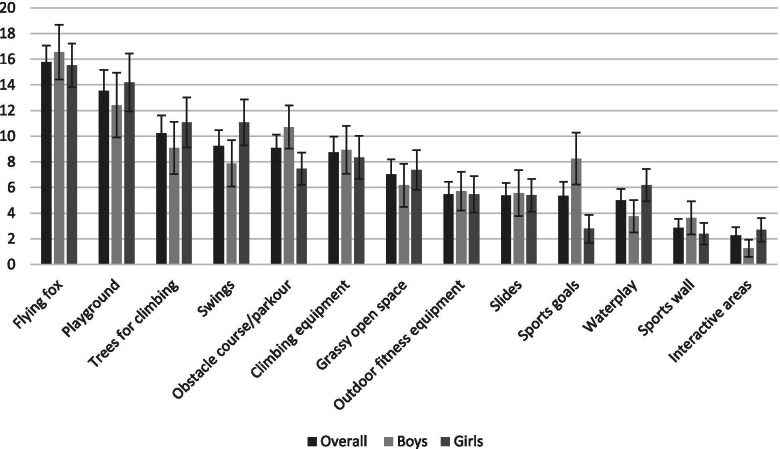
Relative importance scores for encouraging physical activity among children

Overall, the two most important park features influencing choice were a flying fox (e.g., zip line cable between two points) (15.8%; 95%CI = 14.5, 17.1) and a playground (13.5%; 95%CI = 11.9, 15.2), followed by trees for climbing (10.2%; 95%CI = 8.9, 11.6) and swings (9.2%; 95%CI = 8.0, 10.5) (Fig. 2). Interactive areas were the least important feature (2.3%; 95%CI = 1.6, 2.9).

A few differences were observed by gender. Swings was third most important for girls (11.09, 95%CI = 9.3, 12.9), whereas it was seventh most important for boys (7.9, 95%CI = 6.1, 9.7). Water play areas was eighth most important for girls (6.2, 95%CI = 4.9, 7.5) and the 11th most important for boys (3.8, 95%CI = 2.5, 5.0). Obstacle course/parkour areas was third most important for boys (10.7, 95%CI = 9.0, 12.4) and sixth most important for girls (7.5, 95%CI = 6.6, 8.7) and sports goals was sixth most important for boys (8.3, 95%CI = 6.2, 10.3) and 11th most important for girls (2.8, 95%CI = 1.7, 3.9).

## Discussion

This study used ACBC analyses to identify the relative importance of specific park features for influencing choice of park for encouraging physical activity among children. In general, for every feature examined, children preferred the existence of a feature over the absence of a feature, and they preferred the bigger or more challenging level described. For example, a long flying fox was preferred over a short flying fox. Overall, across all features in the ‘tournament’, the three features that were the most important driver of choice for a park that would encourage them to be active were a flying fox, a playground, and trees for climbing. For girls, however, swings had the third highest importance score and for boys an obstacle course/parkour area had the third highest importance score. Therefore, to cater for both boys and girls, long flying foxes, a large adventure playground, lots of trees for climbing, large swings, and obstacle course/parkour areas should also be prioritised to encourage active park use.

Overall, our findings suggest that parks equipped with a diversity of play elements are more likely to appeal to children as a park that would encourage them to engage in park-based physical activity. This supports research from the US that found that the more play elements present in a playground, the more visitors of all ages visited the playground and engaged in park-based physical activity [46]. When planning the playground equipment, it is critical to consider the type of equipment. For example, for both the overall sample and boys and girls, large round swings had the highest part-worth utility scores and therefore, planners should consider the specific type of swing that is installed. Children also appeared to consistently prefer feature levels with the highest level of physical challenge (e.g., long flying fox, large adventure playground, large round swings). This is consistent with previous research that highlighted children’s preference for physical challenge during park play [30, 36]. For example, in a recent qualitative study, children stated that the park features they liked the most were the play elements with risk and challenge and the park features they disliked were the elements that were too small or ‘boring’ [36]. Previous research has also identified that elements of physical challenge are necessary to stimulate and support park-based physical activity among adolescents [31, 35]. A systematic review also found ‘risky’ outdoor play to be positively associated with a variety of health behaviours among children [47]. When planning and renewing parks, stakeholders should give careful consideration to equipping parks and playgrounds with elements that are challenging and adventurous to support physical activity among youth.

It is unsurprising that sports goals were of higher importance for boys (6th) than for girls (11th), as boys are more likely to play team sports such as soccer or basketball and girls are more likely to participate in less team orientated activities such as dancing and gymnastics [48]. Previous research has also highlighted boys’ preference for sports-related activities [18, 23]. Obstacle course/parkour areas also had a higher importance score for boys (3rd) than for girls (6th), although this feature had the fifth highest importance score overall. Obstacle courses/parkour equipment is relatively new in parks in Australia and further research is required to understand specific details on the type of obstacle/parkour equipment that may promote sustained and active use among children.

For the overall sample, water play areas, sports walls and interactive areas had the three lowest importance scores of the 13 features examined in this sample of children. This was generally consistent for both boys and girls; however, water play areas had a higher importance score (8th) among girls than boys (11th). This does not mean that these features are not important; it just shows that of the 13 included features that they had the lowest importance. However, previous research has reported lower physical activity levels among children using water play areas compared to playground areas [21]. Interestingly, in our previous research, children ranked interactive areas highly for encouraging visitation and park-based social interaction [38]. Therefore, although in the present study they were not found to be as important as other features for driving choice for a park that would encourage physical activity, interactive areas may support and promote park visitation but not necessarily physical activity. These types of digital installations are a relatively new feature in parks, so future research is necessary to explore the types of equipment that may be most appealing for children as well as other population groups.

Our findings highlight the need to consider the specific needs of children, and the differential needs of boys and girls, when designing parks as their preferences are different to those of older adults (under review) and adolescents (under review). For example, two studies that also used ACBC analysis found the three most important drivers of preference for parks that would encourage park-based physical activity among adolescents were the presence of sports courts, grassy open space, and outdoor fitness equipment (under review) and among older adults were walking paths, shady trees, and a peaceful and relaxed setting (under review). This confirms the need to consider all age groups when planning parks; as well as inter-generational needs, as previous research has shown that a large percentage of older adults visit parks with their grandchildren [37].

## Strengths and limitations

To our knowledge, this is the first study to use ACBC analysis to examine this research question among children. Obtaining input from children directly is critical to ensure that park design meets their specific needs and analysis by gender made it possible to determine if preferences for particular features were more or less important for boys and girls. However, it is important to acknowledge the following limitations and considerations. The selected features were based on results from walk-along interviews in urban parks with children [36] and children’s ratings of digital images [38]; however, it is possible that other park features may be more/less important than the features included. The inclusion of children attending schools located in low, mid and high SES areas, who were both regular and irregular park users, are further strengths of this study as it enabled us to obtain information from participants living in neighbourhoods with diverse socio-demographic characteristics and with varied park experiences. Future studies should consider including children living in non-urban areas as park features have been shown to differ in urban versus rural parks [49], and children in rural areas may have different preferences. Future studies may also wish to examine preferences according to individual level SES. Most children in the current study reported visiting a park at least once per week (55%) and (80%) reported usually engaging in MVPA during their park visits, so it would also be valuable for future research to identify if specific park features would encourage children who visit less regularly or who are usually less active during their park visits to be more active when at the park. Although challenging [46], ideally future research should also seek opportunities for natural experimental studies to examine the impact of incorporating these findings in park design and examining the impact of these changes on children’s park-use behaviour.

The methodology employed is a novel approach to obtaining information about drivers of preference for park features over a series of choice tasks. While ACBC survey tasks can be considered complex, the in-class methodology with research supervision helped to ensure successful completion with children 8-12 years as they could ask questions and receive assistance at the time of completing the survey. Features were described with a written description, which is consistent with previous conjoint analysis studies [31, 50]; however, it is possible that the children may have had varied interpretations of the written descriptions. To help minimise this potential limitation, examples of the features presented in the ACBC survey were presented as images on handouts during survey completion. Future studies may consider the use of images of park features within the ACBC survey. Finally, the focus of this study was on in-park features. Future studies should also examine the relative importance of other factors external to the park that influence park visits, such as proximity to home, accessibility, and transportation.

## Conclusion

This study provides much-needed practical evidence and insights to inform the planning and design of future parks (re)developments to support active park use among children based on their preferences. To ensure parks are appealing as a setting that encourages children to engage in physical activity during their park visits, the findings from this study suggest that policymakers should prioritise the inclusion of a long flying fox, large adventure playgrounds, lots of trees for climbing, large round swings, obstacle courses/parkour areas and large climbing equipment. The next step is to examine the impact of incorporating these findings in park design and examining the impact of these changes on children’s park-use behaviour [39]. Future planning decisions should consider park feature preferences as well as other factors that impact park use such as accessibility, and availability.

## Supplementary Information

**Additional file 1.** Examples of Adaptive Choice Based Conjoint Analysis survey questions.

## Data Availability

The dataset used and/or analysed during the current study are available from the corresponding author on reasonable request.
