# Supplementary material for: Understanding children’s preference for park features that encourage physical activity: an adaptive choice based conjoint analysis
Source: Int J Behav Nutr Phys Act. 2021 Oct 9;18:133. doi: 10.1186/s12966-021-01203-x (PMC8501594; doi:10.1186/s12966-021-01203-x)

**Examples of Adaptive Choice Based Conjoint Analysis survey questions**

**Example of question selecting preferred levels:**


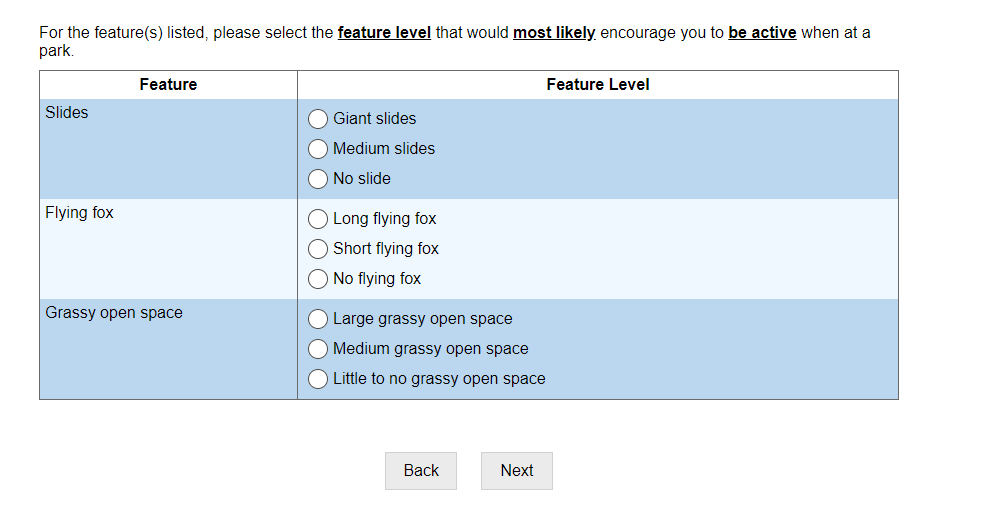


**Example of a screener question**:


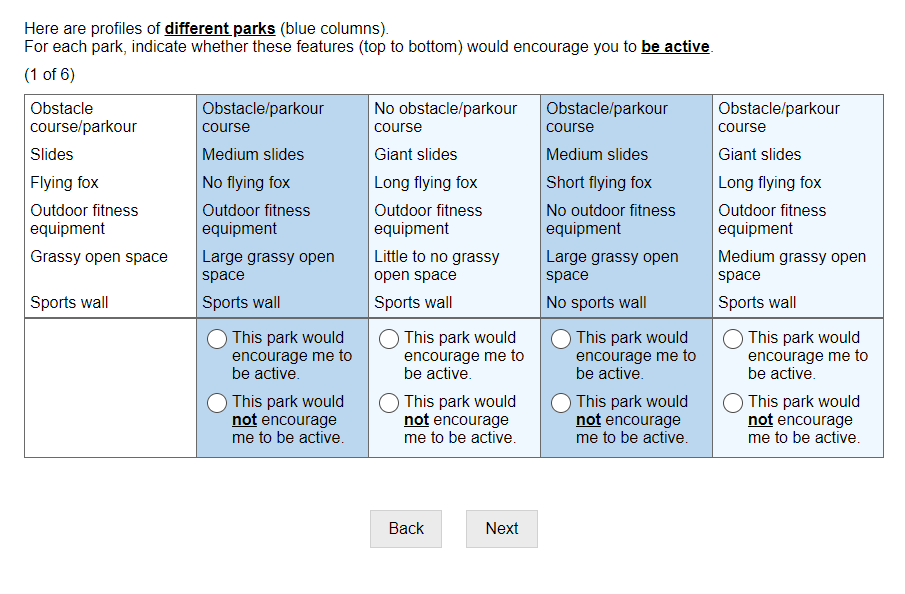


**Example of question asking if any feature levels are totally unacceptable:**


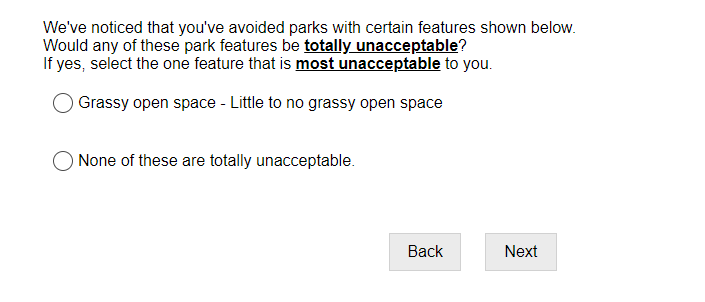


**Example of a choice task:**


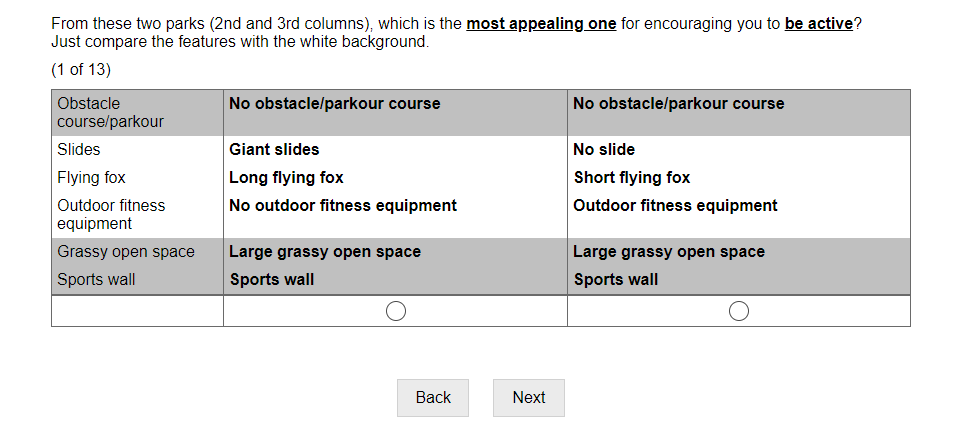

Supplement: Supplementary file 1 — Additional file 1. Examples of Adaptive Choice Based Conjoint Analysis survey questions. [file 12966_2021_1203_MOESM1_ESM.docx]
